# Large-sized pleomorphic adenoma of the cheek treated with Nd:Yag laser: report of a case and review of the literature

**DOI:** 10.4317/jced.56274

**Published:** 2020-09-01

**Authors:** Federica Veneri, Marco Meleti, Luigi Corcione, Elena Bardellini, Alessandra Majorana, Paolo Vescovi

**Affiliations:** 1 DDS. Postgraduate student of the Pediatric Dentistry Program - Department of Medical and Surgical Specialties, Radiological Sciences and Public Health, Dental School, University of Brescia, Italy; 2 DDS, PhD. Researcher in Oral Medicine and Surgery – Centro Universitario di Odontoiatria – Department of Medicine and Surgery – University of Parma, Italy; 3 MD. Department of Human Histopathology, Academic Hospital of Parma, Italy; 4 DDS, PhD. Associate Professor - Department of Medical and Surgical Specialties, Radiological Sciences and Public Health, Dental School, University of Brescia, Italy; 5 MD, DDS. Full Professor – Director of the Postgraduate Pediatric Dentistry Program - Department of Medical and Surgical Specialties, Radiological Sciences and Public Health, Dental School, University of Brescia, Italy; 6 DDS, PhD. Associate Professor in Oral Medicine and Surgery - Centro Universitario di Odontoiatria - Department of Medicine and Surgery, University of Parma, Italy

## Abstract

Pleomorphic adenoma (PA) mostly involves parotid glands, while extra-parotid localizations are relatively uncommon. Particularly, PAs of the cheek minor salivary glands with a size larger than 4 cm are exceedingly rare, with only few cases reported. Surgical treatment of PA usually consists in radical excision. However, despite a presumptive radicality, recurrences, sometimes followed by malignant transformation, may occur. Here we report a case of a large-sized (6 cm) PA of the cheek minor salivary glands in a 70 year-old female patient, successfully treated through a conservative approach, based on the use of Nd:YAG Laser (λ=1064 nm). No recurrences were observed after a 2-year follow-up. A concise review of the literature, describing the features of 14 cases is also provided. Advantages of laser treatment include a precise cut, reduction of trauma on surrounding tissues, the possibility of a very good intraoperative hemostasis. Such features may sometimes allow to avoid general anesthesia, even for removal of big lesions. Post-operative course, in terms of pain and swelling, is usually better for intervention performed with laser, when compared to traditional surgery.

** Key words:**Oral surgery, oral pathology, pleomorphic adenoma, laser surgery, minor salivary glands, salivary glands tumors.

## Introduction

Pleomorphic adenoma (PA) is the most common benign neoplasm of salivary glands, accounting approximately for 70% of all salivary neoplasms. PA occurs between the 3rd and the 6th decade of life with a slight female predilection. Only 8% of PAs affect minor salivary glands, the most common intra-oral sites being the palate (60-65%), followed by the lip (10%) and buccal mucosa. PAs affecting minor salivary glands of the cheek are very rare, accounting for less than 1% of all PAs (1). Surgical excision with clear margins is the treatment of choice. To the best of our knowledge, extra-parotid PA of the minor salivary glands within the cheek and mucobuccal fold has been described only in a few cases, thus representing a very unusual finding. (1-14). Here we report a rare case of a large-sized PA arising from minor salivary glands of the cheek, successfully treated through Neodimium: Yttrium Aluminium Garnet (Nd:YAG) Laser, altogether with a concise review of the literature of the last 30 years, focused on PAs of minor salivary glands larger than 1 cm (1990 to 2019) (Table 1,  Table 1 cont.).

**Table 1:**
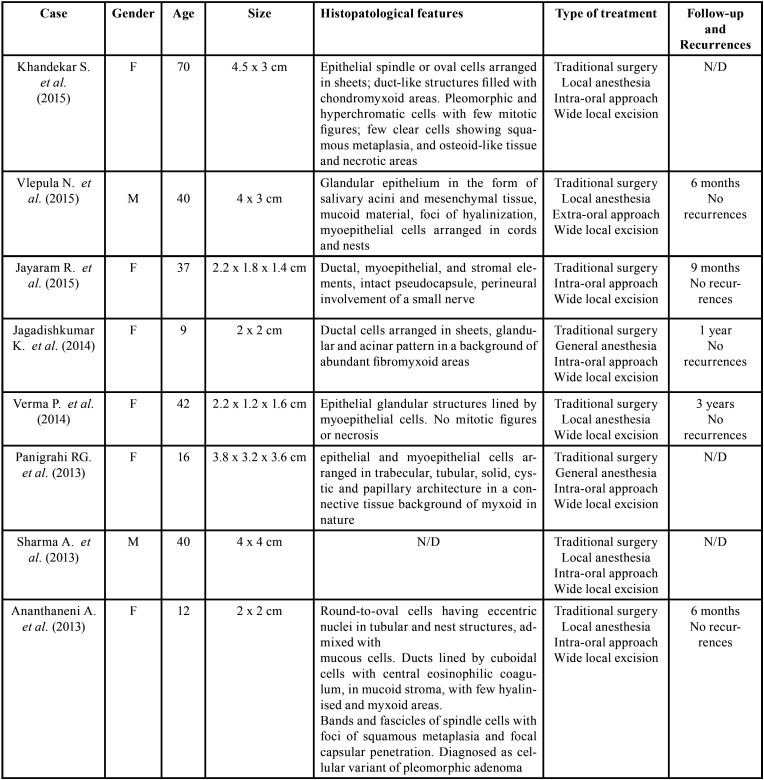
Review of the literature: clinico-pathological features of 14 cases of pleomorphic adenoma of minor salivary glands of the cheek and mucobuccal fold, larger than 1 cm, identified from January 1990 to July 2019.

**Table 1 cont.**
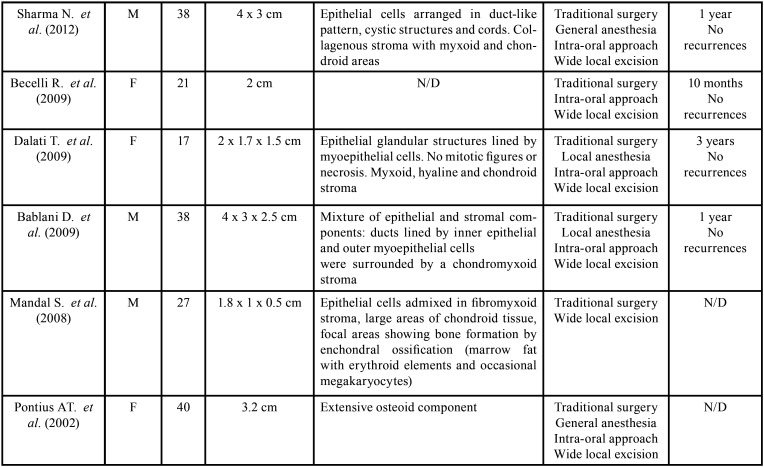
Review of the literature: clinico-pathological features of 14 cases of pleomorphic adenoma of minor salivary glands of the cheek and mucobuccal fold, larger than 1 cm, identified from January 1990 to July 2019.

## Case Report

**Figure 1:**
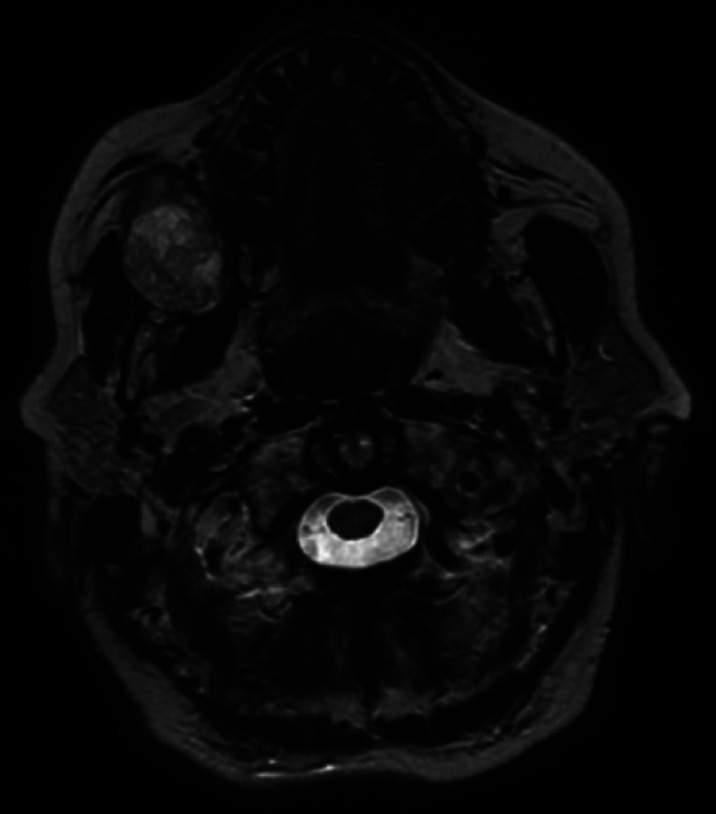
MRI. Axial T2-weighted images showing a mass with well-defined margins, smooth borders, slight inhomogeneous pattern and high-intensity signal; the mass seems encapsulated.

A 70-year-old female patient was referred for a painless, slow-growing swelling within the right cheek, which was present since the last 15 years and significantly increased during the last year. Extraoral clinical examination revealed asymmetry of the face caused by the presence of a mobile, hard-elastic mass. Intraoral evaluation showed a swelling covered by intact mucosa, with a dimension on the major size of approximately 5 cm. At magnetic resonance imaging (MRI), the mass showed well-defined margins (most likely associated to the presence of a capsule), smooth borders, inhomogeneous aspect and a high-intensity T2-weighted signal. The mass was adherent to Bichat’s fat pad, delimited by the Stensen’s duct and masseter muscle, the upper fornix and lower fornix posteriorly and the facial artery anteriorly (Fig. 1).

**Figure 2:**
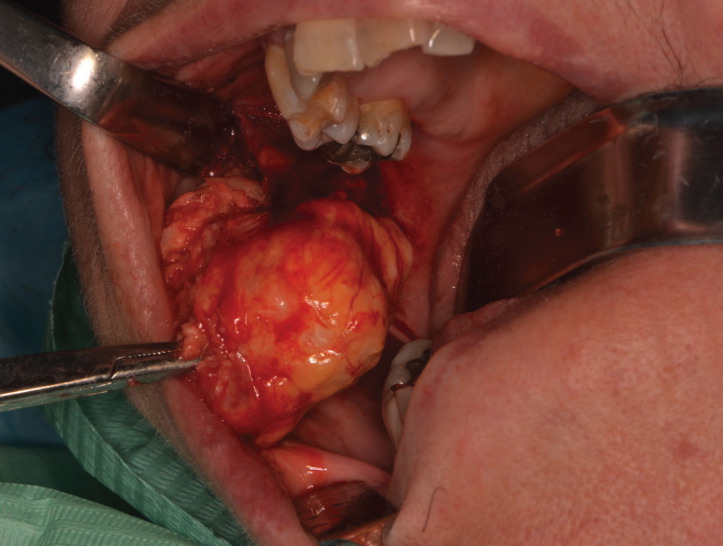
Surgical excision. Intra-oral incision of the overlying mucosa performed with Nd:YAG laser (Fidelis Plus®, Fotona – Slovenia - 3.5W, 60Hz, PD 488,281W/cm2) and isolation of the tumor from the surrounding tissues.

On the basis of a presumptive diagnosis of a benign tumor of minor salivary glands or of connective tissue, surgical excision trough Nd:YAG laser (λ=1064 nm, Fidelis Plus®, Fotona – Slovenia - 3.5W, 60Hz, PD 488,281W/cm2 ) was performed under local anesthesia (Fig. 2).

**Figure 3:**
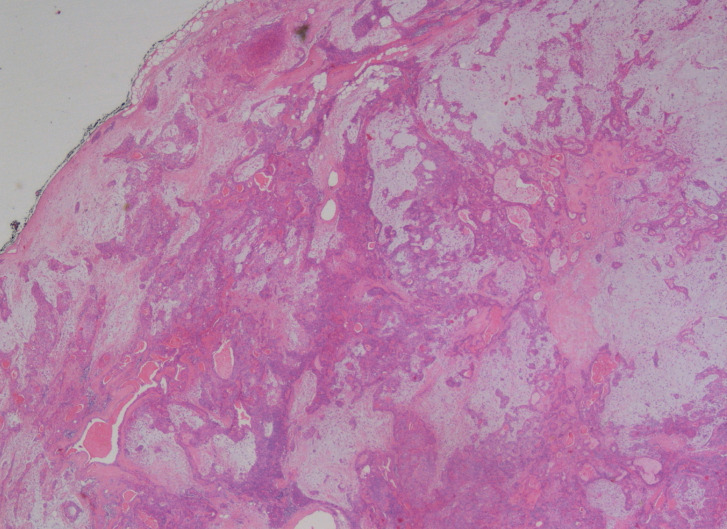
Microscopic view. pleomorphic adenoma showing a mixture of glandular epithelium and myoepithelial cells within a mesenchyme-like background, with a mixed mucoid, myxoid or chondroid appearance (Haematoxilyn and Eosin staining – 10X magnification).

At macroscopic examination the mass measured 6 x 3.5 x 3 cm, with a whitish and glistening cut surface and multiple translucent cystic areas. Histopathological examination showed a mixture of epithelial and stromal tissue. Ducts lined by inner epithelial and outer myoepithelial cells were surrounded by a chondromyxoid stroma, consistent with pleomorphic adenoma. Being the tumor mass not associated with the parotid gland or duct, it was considered to be of minor salivary gland origin. The microscopic examination therefore confirmed the diagnosis of PA; all margins were free from neoplastic infiltration (Fig. 3). No pain or swelling were present after 2 weeks. No clinical and MRI signs of recurrence were noticed after 2-year follow-up.

## Discussion

Most of patients with PAs of minor salivary glands complain for the presence of a small, painless, nodule which slowly increases in size, sometimes showing intermittent or accelerating growth (3). Most cases are asymptomatic and there is usually a long period of time (ranging from few days up to 15 years) between the appearance of the lesion and the diagnosis (12). Differential diagnosis of PA of cheek mucosa should include odontogenic and non-odontogenic buccal abscesses, salivary glands infections and other inflammatory conditions such as sialolithiasis. Further non-oncologic causes of swelling include dermoid and sebaceous cysts as well as reactive lymph nodes. Among neoplasms, diagnostic hypotheses should include benign tumors such as lipomas, neurofibromas and Warthin’s tumor as well as malignancies like mucoepidermoid and adenoid cystic carcinomas, polymorphous adenocarcinoma and carcinoma ex pleomorphic adenoma (9).

While Velpula and other authors performed ultrasonography, fine needle aspiration cytology (FNAC) and/or computed tomography (CT) prior to MRI, we performed MRI (1,2,4,14). Being the clinical presentation strongly suggestive for a benign tumor of soft tissues, MRI was considered to be appropriate as first line imaging investigation, thus limiting invasiveness and unnecessary exposure to radiation (10). At MRI, benign salivary gland tumors typically show well-defined borders and hyperintense signal on T2-weighted images. On the contrary, ill-defined borders, invasion into surrounding tissues, low T2 signal, heterogeneous enhancement, cystic changes and central necrosis are suggestive of malignancy (1,10). In the present case, MRI features were consistent with a benign tumor, probably of minor salivary gland origin. In fact, axial T2-weighted images showed well-defined and smooth borders and high-intensity signal. A slight inhomogeneous aspect could also be observed, probably due to the large size of the mass and to a degeneration induced by its long persistence. These findings were similar to the majority of the cases reviewed, where imaging information was available (1,2,7,10,14).

From the pathologic macroscopic point of view, PAs of minor salivary glands are often grossly encapsulated and they usually range from 1 to 4 cm in size (2). Larger lesions may show necrotic or cystic areas. Their consistency varies from hard to rubbery to soft, sometimes fluctuant, swellings. The cut surface of the mass is solid and the color may vary from gray blue, yellow to brown. Gritty, gelatinous or glistening areas may be present when there is a cartilaginous or myxochondroid differentiation (4). Mandal *et al*. reported a peculiar case of PA showing bone and bone marrow formation within chondroid tissue (13). In the present case, both epithelial and myoepithelial cells, typical of PAs, were present; such a finding, may be useful, to some extent, to rule out mucoepidermoid carcinoma. The absence of perineural invasion and mitotic Figures reduced the chances of polymorphic adenocarcinoma and carcinoma ex pleomorphic adenoma. Nevertheless, Jayaram *et al.* reported a case of perineural involvement of a small nerve associated with a benign PA (5).

PAs might sometimes recur, either due to seeding or inadequate removal (1,12). PA does not spread through the lymphatic or blood system and does not have the ability to induce locoregional or distant metastasis. However, in few cases a malignant epithelial tumor has been documented within the context of a PA, being known as carcinoma ex PA (12).

Moreover, PAs show sometimes signs of extracapsular spread at postoperative histopathologic examination (9). Such a feature may account for some of the recurrences observed in cases of presumptive radical excision (12). On the basis of the impossibility to precisely assess the presence of malignant cells or microscopic extracapsular spread, the preferred treatment for PA consists in wide local excision (0.5 cm free margins) and follow-up for at least 3-4 years (4). This procedure was, in fact, performed in the totality of the reviewed cases, where no recurrences were reported.

In our case, Nd:YAG laser was chosen for its precise and minimally invasive cut, which allows radical excision in most cases. Nd:YAG laser wavelength also provides a powerful haemostatic activity (15).

The energy transferred by the laser beam to biological tissues results in tissue heating. The mean thickness of epithelial, stromal and vascular changes induced by Nd:YAG laser, as detected at microscopic evaluation, is 363.7, 459.1 and 169.85 micron, respectively (15). It has been demonstrated that Nd:YAG laser causes gross changes in small mucosa specimens (less than 7 mm in the main size), independently from laser settings. On the other hand, the heat-induced damage is negligible in specimen larger than 7 mm. In these cases, it is usually possible to perform a precise histological evaluation of the margins (15). We also hypothesize that another advantage of laser surgery is the carbonization of possible residual PA cells on the excision margins. All cheek PA cases, described in the literature considered here, underwent traditional surgery and approximately 30% of them (4 of the cases, where the information was available) was carried out under general anesthesia (6,7,10,14).

Nd:YAG laser also shows a strong antimicrobial activity, which reduces the need for antibiotics and the rate of infectious complication, even in cases of second intention wound healing (15). In the present case, the use of laser gave the possibility to perform the surgical intervention within an outpatient setting, allowing to save time and economic resources, if compared with an intervention performed under general anesthesia followed by hospitalization.
